# A novel simple experimental model for low-osmolar contrast-induced acute kidney injury using different definitions based on the levels of serum creatinine and cystatin C

**DOI:** 10.1186/s12882-019-1436-5

**Published:** 2019-07-04

**Authors:** Yuan-hui Liu, Jin-hua Xue, Deng-xuan Wu, Wei-jie Bei, Kun Wang, Yong Liu, Ji-yan Chen, Ning Tan

**Affiliations:** 1 Department of Cardiology, Guangdong Cardiovascular Institute, Guangdong Provincial Key Laboratory of Coronary Heart Disease Prevention, Guangdong Provincial People’s Hospital, Guangdong Academy of Medical Sciences, Guangzhou, 510100 Guangdong China; 2 Department of Physiology, School of Basic Medical Sciences, Gannan Medical University, Ganzhou, 341000 China

**Keywords:** Contrast, Acute kidney injury, Model

## Abstract

**Background:**

It remained lack of a kind of contrast-induced acute kidney injury (CI-AKI) model which was widely used in clinical practice and comparable to CI-AKI in humans.

**Methods:**

Fifty Sprague-Dawley rats were divided into five groups of 10 rats each: (1) sham group (normal saline [NS] + NS); (2) NS plus low osmolality contrast medium (CM15) (NS + CM15); (3) furosemide (FM) plus NS (FM + NS); (4) FM + CM10; and (5) FM + CM15.We measured the levels of serum creatinine (SCr), cystatin C (cys-C) and histopathological scores of kidney tissues.

**Results:**

SCr level in the FM + CM15 group were significantly increased after CM exposure compared with baseline levels (32.9 ± 4.57 vs. 158.7 ± 14.48 μmol/L, *p* < 0.001). Minor changes were found about the SCr levels between the pre- and post-exposure CM or NS treatment in the other groups. Additionally, the cys-C levels after CM exposure were increased compared with pretreatment levels in the FM + CM15 group (0.08 ± 0.03 vs. 0.18 ± 0.05 mg/L, *p* < 0.001). Minor changes were noted in the FM + NS group before and after NS administration. Only rats in the FM + CM15 group developed CI-AKI with the definitions of SCr or cys-C. Comparing to the FM + NS group, the histopathological scores were significantly increased in the FM + CM15 group.

**Conclusions:**

A simple and reliable animal model for low osmolality contrast medium-induced AKI was established, which is similar to clinical CI-AKI based on different definitions for AKI.

## Background

Contrast-induced acute kidney injury (CI-AKI) is a serious common complication in patients after contrast medium (CM) exposure during cardiac catheterization and computed tomography. CI-AKI increases the length of hospital stay, health care cost, and rate of in-hospital complications, including a mortality rate of approximately 20%, and patients may become predisposed to long-term loss of kidney function [1–4]. However, other than preprocedural hydration and limiting the CM volume, few strategies have been proven effective for preventing CI-AKI [5]. Although an extensive amount of work has been performed during the past 20 years, the pathophysiology of CI-AKI remains obscure [6]. The mechanisms involved in CI-AKI play important roles in understanding and preventing CI-AKI. Nevertheless, progress in understanding this pathology seems to have been hampered by the lack of a reliable and reproducible experimental model for CI-AKI.

Unfortunately, most previous CI-AKI models are not appropriate given that they were induced in rats by the intravenous injection of a high osmolality CM, in addition to reagents that inhibit prostaglandin (indomethacin) and nitric oxide synthesis [7–9], which are associated with the greatest risk of developing CI-AKI and are not used in clinical practice. In contrast, a low or iso-osmolality CM is widely used in clinical settings. Nevertheless, few studies have established an experimental model for CI-AKI based on low osmolality CM (LOCM) without other nephrotoxic drugs, that would be comparable to clinical CI-AKI in humans. In addition, more novel biomarkers for detecting CI-AKI have been reported. Cystatin C (cys-C) provides increased sensitivity with equivalent specificity for detecting CI-AKI compared to serum creatinine (SCr). Therefore, the present study aimed to develop an experimental model for LOCM-induced AKI with different definitions for AKI based on SCr and cys-C to develop new mechanisms or strategies for preventing CI-AKI in the future.

## Methods

### Ethic statements

The experimental procedures were finished by the guiding of the Care and Use of Laboratory Animals, which was published by the US National Institutes of Health And it was also approved by the Institutional Review Board of the Blinded and Hospital, Guangdong, China. Adult male Sprague-Dawley rats were purchased from the Laboratory Animal Science Department of the Blinded (License No. SYXK (Yue) 2010–0056).

### Animals

We used adult male Sprague-Dawley rats aged 8–10 weeks old (190–230 g). The rats were maintained in standard housing facilities with a 12 h light/12 h dark cycle at 24 ± 1 °C and 45 ± 5% humidity. At the beginning of the study, the rats were acclimatized for 7 days. The experimental procedures were performed by the guiding of our institutional and national guidelines for animal research.

### Experimental design and the contrast medium

The rats were randomly allocated into the following five groups (*n* = 10 for each group and each experiment): (1) sham (normal saline [NS] + NS), (2) NS plus contrast medium (CM15) (NS + CM15), (3) furosemide (FM) plus NS (FM + NS), (4) FM + CM10, and (5) FM + CM15.

After acclimatization for 7 days, and 6 h before CM administration, groups FM + NS, FM + CM_10_ and FM + CM_15_ received furosemide (Harvest Pharmaceutical Co., GuangZhou, China), which was injected intramuscularly (10 mL/kg). The other groups received the same volume of saline. Then, all the groups had restricted access to water for 6 h. After 6 h, groups NS + CM_15_ and FM + CM_15_ groups received LOCM (350 mg I/mL, Bayer, Guangzhou, China) in the tail vein at a dose of 15 mL/kg, whereas the FM + CM_10_ group received 10 mL/kg of LOCM for under ether anesthesia. The NS + NS, and FM + NS groups received the same dose of normal saline. Subsequently, all the rats were given unlimited access to standard rat chow and water. The rats were weighed before the furosemide and LOCM injections and at 24 h after CM administration. After 24 h following CM administration, rat blood was sampled to determine the SCr levels, and rats were sacrificed by decapitation. Their kidneys were removed for histological analyses.

### Renal function parameters

Approximately 1.3 mL of blood was collected from the tail vein and was placed into a plain tube before LOCM or normal saline was injected under ether anesthesia. The blood was to be cloted at least 45 min. Serum was collected after centrifugation at 2000×g for 10 min and was analyzed for the SCr and cys-C levels. The final blood sample was analyzed at the end of the study (24 h) in the same manner. The SCr and cys-C measurements were performed using a biochemical automatic analyzer.

### Histological evaluation of kidneys

Histological evaluation of kidneys was performed in accordance with previous researches [10, 11]. The kidneys were excised and cut from the top to the bottom after 24 h. Tissues were fixed in 10% neutral-buffered formalin and dehydrated in a graded series of alcohols. Then, tissues were deparaffinized in xylenes and were paraffin-embedded. Subsequently, 5-μm thick sections were cut from the paraffin blocks and were routinely dewaxed and hydrated. The slices were stained with hematoxylin, rinsed with water, differentiated with 1% hydrochloric acid alcohol, stained with eosin for 1 min and rinsed again with water. Finally, the slices were dehydrated with alcohol, deparaffinized in xylene again and mounted with cover slips. We performed the histopathological evaluation of the kidney glomeruli, tubules, interstitium, and arteries through a board-certified veterinary pathologist. And the process was blinded to the experimental groups. The extent of injury was based on the following criteria: no injury; mild; moderate; severe; and very severe (0; < 25%; < 50%; < 75%; > 75%, respectively) [12].

### Definitions of CI-AKI

The CI-AKI was defined according to one of the following definitions: an absolute increase in SCr levels of ≥44.2 μmol/L or a relative increase of ≥25% from baseline within 48–72 h after CM exposure [13]; and an increase in the cys-C concentration of 10% greater than the baseline value at 24 h after the administration of CM [14].

### Statistical analysis

Continuous variables are expressed as the mean ± standard deviation or as their median (inter quartile range). Student’s t-test or one-way analysis of variance was performed to determine differences among the groups. Categorical variables are reported as absolute values and percentages and they were analyzed using the chi-square test or Fisher’s exact test. SAS version 9.2 (SAS Institute, Cary, NC, USA) was used to analysis. All the probability values were two-tailed and the statistical significance was defined as *p* < 0.05.

## Results

### Body weights

The body weight was significantly reduced in all three groups (FM + NS, FM + CM10, and FM + CM15) that were administered FM for dehydration. A limited number of changes were noted in the other groups that received NS. In addition, at the end of the experiment, the rat’s body weights in the groups that were given CM and FM were significantly reduced compared with the other groups.

### SCr and cys-C concentrations

The concentrations of SCr in each group before and after CM or NS exposure are presented in Table 1. The SCr levels in the NS + NS, NS + CM15, FM + NS and FM + CM10 groups were minimally changed between the pre- and post CM or NS exposure. However, in the FM + CM15 group, the SCr concentration was significantly increased after CM exposure compared with baseline levels (32.9 ± 4.57 vs. 158.7 ± 14.48 umol/L, *p* < 0.001). In addition, to confirm the change in the SCr of the FM + CM15 group, we also measured the levels of cys-C in FM + CM15 and FM + NS groups to demonstrate the effect of CM15 on renal function. The cys-C levels were also increased after CM exposure compared with baseline in the FM + CM15 group (0.08 ± 0.03 vs. 0.18 ± 0.05 mg/L, *p* < 0.001). However, no significant difference was noted in the FM + NS, NS + NS and NS + CM15 group before and after NS administration (0.07 ± 0.01 vs. 0.06 ± 0.01 mg/L; 0.03 ± 0.01 vs. 0.04 ± 0.01 mg/L; 0.04 ± 0.01 vs. 0.04 ± 0.01 mg/L; all *p*>0.05).

**Table 1:** Changes of serum creatinine and body weight in each group

Parameters	SCr (umol/L)		Changes	Body weight (mg)				
Times	Pre CM or NS	Post CM or NS	Post-Pre	Baseline	6 h after FM or NS	6 h-baseline	24 h after CM or NS	24 h–6 h
NS + NS	17.6 ± 2.07	20.5 ± 2.64	1.1 ± 1.5	207.9 ± 6.30	206.8 ± 6.18	− 3.0 ± 2.6	204.5 ± 7.72	−1.7 ± 3.2
NS + CM15	17.6 ± 2.07	18.7 ± 1.57	2.9 ± 2.7	207.3 ± 9.54	204.20 ± 9.09	−1.2 ± 4.2	202.54 ± 7.94	− 2.3 ± 6.2
FM + NS	36.2 ± 6.27	23.2 ± 2.78	−13.0 ± 4.3	203.80 ± 6.1	185.2 ± 6.0	−19.0 ± 3.0	198.03 ± 15.23	0.4 ± 10.6
FM + CM10	38.6 ± 6.85	27.1 ± 7.19	−11.5 ± 8.5	203.9 ± 9.60	184.90 ± 8.70	−20.4 ± 1.8	185.30 ± 8.88	− 8.7 ± 11.1
FM + CM15	32.9 ± 4.57	158.7 ± 14.48	125.8 ± 17.0	202.6 ± 8.20	182.2 ± 7.30	− 18.6 ± 4.3	173.50 ± 12.62	12.8 ± 10.6

Abbreviation: *NS* normal saline, *CM* contrast medium, *FM* furosemide

### Incidence of CI-AKI based on the SCr and Cys-c

CI-AKI was not induced based on the definition of SCr or cys-C in the NS + NS, NS + CM15, FM + NS and FM + CM10 groups. Nevertheless, based on the definitions for SCr and cys-C, CI-AKI developed in the FM + CM15 group.

### Histopathological results

**Fig. 1:**
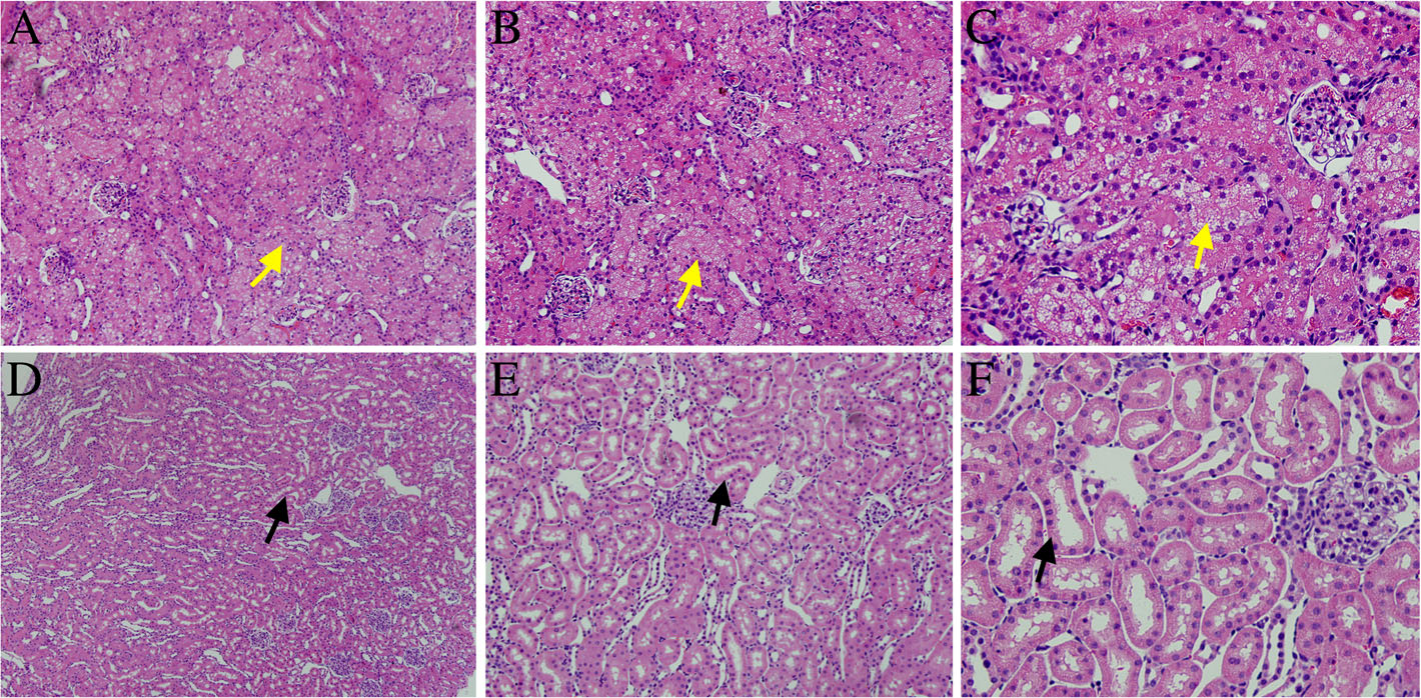
Representative photomicrographs of the tubular cell injury in rat kidney tissue sections from the furosemide (FM) + normal saline and FM + contrast medium 15 groups. Original magnifications:× 100(**a** and **d**), × 200(**b** and **e**), and × 400 (**c** and **f**). Hematoxylin and eosin staining. Calibration bar = 20 μm. (:the lumen and structure of renal tubular was normal.:The lumen of renal tubular was changed into small, even occlusion. And there is severe detachment and foamy degeneration of the tubular cells

The histopathological scores of the FM + NS and FM + CM15 groups are presented in Fig. 1. Glomerular sclerosis and interstitial fibrosis were not observed in any of the groups. Noticeable detachment or foamy degeneration of tubular cells was not observed in the FM + NS group (histologic scoring: 0.40 ± 0.52; Fig. 1d-f). However, there was severe detachment and foamy degeneration of the tubular cells was noted in the FM + CM15 group (Fig. 1a-c), and the histologic scores were significantly increased compared with the FM + NS group (3.3 ± 0.82, *p* < 0.001).

## Discussion

The present study established a novel simple CI-AKI model based on LOCM and different definitions of CI-AKI, which included a novel biomarker for CI-AKI.

CI-AKI remains a serious complication in patients exposed to CM, and it is strongly associated with poor short- and long- term outcomes. Clinically relevant animal models of CI-AKI are useful for further understanding the mechanisms involved, identifying novel biomarkers, evaluating potential differences among different CM types, and identifying new strategies for preventing CI-AKI.

However, most of the previous CI-AKI animal models involved rats, and used indomethacin (10 mg/kg; intravenously, [IV]), followed by Nw-nitro- L-arginine methyl ester (L-NAME, 10 mg/kg, IV) after 15 min, and by 6 mL/kg of high-osmolar radiological contrast agent 60% meglumine amidotrizoate injected into the tail vein after additional 15 min [8, 15, 16]. Experimental observations demonstrated that the use of nonsteroidal anti-inflammatory drugs (indomethacin) increased the risk of developing CI-AKI [17], and previous guidelines suggested that patients should be withdrawn from nephrotoxic drugs at least 24 h before CM administration [13]. In addition, contrast nephrotoxicity and the risk of CI-AKI are related to the osmolarity of the CM. It is widely accepted that a high osmolality CM is associated with the greatest risk of developing CI-AKI and is not used in clinical practice in patients who have been exposed to CM [18]. In contrast, the LOCMs were developed and are now widely used. Nevertheless, few animal models have systematically studied LOCM-induced AKI. The present study was performed to demonstrate this issue.

In clinical practice, the incidence of CI-AKI is very low in patients without any risk factors. In contrast, the CI-AKI incidence is significantly increased by up to 50% in patients presenting with multiple cardiovascular risk factors [19]. Therefore, CI-AKI cannot easily be induced by simply exposing healthy animals to CM alone, especially LOCM. Pretreating animals with factors similar to those considered to be risk factors for kidney damage in humans would be helpful for inducing CI-AKI. A previous study demonstrated that the manifestation of CM-induced renal vasoconstriction and oxidative stress, which are the important contributors of CI-AKI development, are the most prominent in a dehydrated animal. Therefore, numerous studies have considered water deprivation for 2–5 days before CM exposure as a routine measure for inducing experimental CI-AKI. Instead of using water deprivation, we used FM before CM exposure to increase the urine output to achieve the same dehydration effect to some extent. We demonstrated that rats pretreated with FM at 6 h before CM exhibited decreased weight and an increased SCr levels, which would make them more prone to developing CI-AKI. Our findings demonstrated that FM with CM induces the development of CI-AKI. Additionally, the hematoxylin and eosin analysis indicated that the tubular cell injury in this group was more severe compared with controls. Studies by Wang et al. [20] and Buyuklu et al. [21] used a CI-AKI model based on LOCM; however, they also used nephrotoxic drugs. Recently, Sun et al. [22] introduced the CI-AKI model, which consisted of dehydration, FM, and Omnipaque. However, they only compared the FM + CM group with the CM group and they demonstrated that the levels of SCr in the former group reach the definitions of CI-AKI. Our findings were similar in that only CM was administered, which cannot induce CI-AKI. However, we found that rats pre-treated with FM also have increased the SCr levels. Therefore, the study by Sun et al. should mimic our present study by adding another group (FM + NS) in order to exclude the effect of FM on increasing SCr levels.

In addition, most of the previous studies established the CI-AKI model according to the definition of CI-AKI as an increase in SCr of ≥25% of the baseline value within 48–72 h [22, 23], which is not often used in clinical practice. The guidelines recommend the following definitions of CI-AKI: an absolute increase in the SCr level of ≥44.2 μmol/L or a relative increase of ≥25% from baseline within 48–72 h after CM exposure. Furthermore, clinical studies demonstrated that an increase in the SCr level of ≥44.2 μmol/L is more sensitive for selectively recognizing more patients with an increased risk of mortality and morbidity. However, SCr increases of ≥25% overestimate CI-AKI by including many patients without the postprocedural relevant deterioration of renal function, and they are affected by a reduced risk of adverse events at follow-up [24, 25]. Therefore, the present study established a CI-AKI model using these two definitions, which are closer to those used in clinical practice.

Although the definition of CI-AKI was based on the SCr concentration, SCr are affected by many status such as muscle catabolism. In addition, its rate of change after the initial insult is low. Moreover, the delayed increase in SCr is a potential reason for overlooking CI-AKI [26] or prolonging the hospital stay in the vast majority of patients who will not develop CI-AKI. However, cys-C is more sensitive than SCr for rapidly detecting acute changes in renal function and achieving a maximum within 24 h after CM exposure [27]. A previous study demonstrated that cys-C seems to be a reliable marker for the early diagnosis and prognosis of CI-AKI [14]. To the best of our knowledge, the current study may be the first to verify the experimental CI-AKI model based on cys-C.

## Limitations

There were several limitations to the present study. Firstly, the dose of the CM was relatively high. However, it was not greater than the maximum safe contrast dose evaluated in a formula by Cigarroa et al. [28] in the humans. Second, we did not obtain the histopathological data or SCr levels at the different time points in order to better understand the development of CI-AKI. Third, we should note that there was a tipping point for AKI in the CM + FM group, which can potentially be detected by various combinations of lower doses of FM and CM. Finally, we did not further investigate the mechanism of CI-AKI mainly through the inflammatory, apoptosis and ischemic/reperfusion.

## Conclusions

In conclusion, a simple and reliable animal model for LOCM-induced AKI was developed for the first time. This model is similar to clinical CI-AKI based on different definitions for AKI.

## Data Availability

N/A.

## Abbreviations

CI-AKI: Contrast-induced acute kidney injury
CM: Contrast medium
cys-C: cystatin C
FM: Furosemide
LOCM: Low osmolality CM
NS: Normal saline
SCr: Serum creatinine
